# Automatic Detection of Brain Metastases in T1-Weighted Construct-Enhanced MRI Using Deep Learning Model

**DOI:** 10.3390/cancers15184443

**Published:** 2023-09-06

**Authors:** Zichun Zhou, Qingtao Qiu, Huiling Liu, Xuanchu Ge, Tengxiang Li, Ligang Xing, Runtao Yang, Yong Yin

**Affiliations:** 1 School of Mechanical, Electrical and Information Engineering, Shandong University, Weihai 264209, China; 2 Department of Radiation Oncology and Physics, Shandong Cancer Hospital and Institute, Shandong First Medical University and Shandong Academy of Medical Sciences, Jinan 250117, China; 3 Laboratory of Image Science and Technology, School of Computer Science and Engineering, Southeast University, Nanjing 210096, China; 4 Department of Oncology, Binzhou People’s Hospital, Binzhou 256610, China; 5 Third Clinical Medical College, Xinjiang Medical University, Urumqi 830011, China

**Keywords:** brain metastasis, computer aided diagnosis, deep learning, YOLOv5

## Abstract

**Simple Summary:**

In this research, we introduced an improved deep learning model for automatic brain metastases detection in MRI. In order to reduce false-positive results while retaining high accuracy, a modified YOLOv5 algorithm with self-attention mechanism is proposed. Our proposed deep learning model showed promising results on the internal testing set, achieving better recall and precision compared to standard algorithms. Furthermore, we also demonstrated the method’s effectiveness and generalizability on the external testing set. The study proved that our proposed framework can be used as a reliable computer-aided diagnosis system for brain metastases detection.

**Abstract:**

As a complication of malignant tumors, brain metastasis (BM) seriously threatens patients’ survival and quality of life. Accurate detection of BM before determining radiation therapy plans is a paramount task. Due to the small size and heterogeneous number of BMs, their manual diagnosis faces enormous challenges. Thus, MRI-based artificial intelligence-assisted BM diagnosis is significant. Most of the existing deep learning (DL) methods for automatic BM detection try to ensure a good trade-off between precision and recall. However, due to the objective factors of the models, higher recall is often accompanied by higher number of false positive results. In real clinical auxiliary diagnosis, radiation oncologists are required to spend much effort to review these false positive results. In order to reduce false positive results while retaining high accuracy, a modified YOLOv5 algorithm is proposed in this paper. First, in order to focus on the important channels of the feature map, we add a convolutional block attention model to the neck structure. Furthermore, an additional prediction head is introduced for detecting small-size BMs. Finally, to distinguish between cerebral vessels and small-size BMs, a Swin transformer block is embedded into the smallest prediction head. With the introduction of the F2-score index to determine the most appropriate confidence threshold, the proposed method achieves a precision of 0.612 and recall of 0.904. Compared with existing methods, our proposed method shows superior performance with fewer false positive results. It is anticipated that the proposed method could reduce the workload of radiation oncologists in real clinical auxiliary diagnosis.

## 1. Introduction

Brain metastasis (BM) is a severe complication of malignant tumors. Approximately 20% of cancer patients develop BM [1]. BM is common among patients with lung cancer, occurring in up to 50% of patients with advanced non-small-cell lung cancer (NSCLC) [2]. This is primarily attributed to advancements in systemic therapies that enable superior control of extracranial neoplasms, thereby improving survival rates and increasing the opportunities for BM development [2,3]. BM developed from NSCLC is linked to poor prognosis, with a median survival of just a few months if left untreated [4]. The symptoms of BM can range from mild headache and cognitive impairment to seizures, focal neurological deficits, and even coma [5]. These symptoms can significantly impact the quality of life and survival period of NSCLC patients. Therefore, early diagnosis and treatment of NSCLC BM is essential to elevate survival rates and improve prognosis.

Radiotherapy is an effective method of treating BM patients and prolonging their survival [6]. Patients with a limited number of BMs can be treated with stereotactic radiosurgery (SRS), which can improve qualities of life and avoid the cognitive decline caused by whole-brain radiation therapy [7,8]. Prior to SRS treatment, accurate detection of BMs, and subsequent delineation of regions of interest (ROI) are necessary. The detection task is mainly performed by experienced radiation oncologists, and remain challenging due to the blurred boundaries and variable quantity of BMs. Therefore, fast and accurate detection of BMs in MRI images with the assistance of deep learning (DL) is of great clinical importance.

In recent years, DL algorithms with a full convolution structure have been proposed for automatic detection of BMs. Zhou et al. [9] used a single-shot detector (SSD) [10] neural network to automatically detect BMs in T1-weighted MRI datasets, achieving a fast and accurate detection capability with a sensitivity of 81% and a positive predictive value (PPV) of 36%. However, this method produced many false positive results that regarded healthy brain tissue as BMs. Using a multi-level feature-fusion (FF) technique, Amemiya et al. [11] developed a modified SSD structure called FF-SSD. Experimental results showed that FF-SSD achieved an overall sensitivity of 86.0% (an increase of 2.2% compared to SSD) and a PPV of 46.8% (an increase of 1.6% compared to SSD). Although FF-SSD can improve the sensitivity and PPV for small-size BMs, it is relatively limited and produces many false positive results. Other approaches rely on 3D convolutional structures to extract the image’s contextual and spatial information for direct detection and segmentation. Using different kernel sizes with the vanilla UNet, Cao et al. [12] compared the asymmetric 3D UNet and proposed asym-UNet to extract the region features of small BMs and the boundary information of large BMs. This study proves that smaller kernels perform better in detection sensitivity while performing worse in PPV. Li et al. [13] developed a two-stage 3D DL model for automatic detection and simultaneous segmentation of BMs. This novel model obtained a sensitivity of 90% and precision of 56%. Although 3D CNN models have achieved excellent performances, they may be difficult to implement on lower-performance computers used in actual clinical practice.

As a DL algorithm, You Only Look Once version 5 (YOLOv5) [14] has achieved excellent precision and recall, and has been applied in the medical image domain for the automatic detection of stroke lesions [15] and polyps [16] as well as the classification of skin cancer [17], among other applications. The effectiveness of these approaches indicates that YOLOv5 can provide image-guided clinical diagnosis and has great potential for new applications in clinical practice. In order to meet the objective demands of BM detection, the existing published works have obtained balance in the ability of their models by adjusting the confidence threshold parameters. However, certain models exhibit limited detection capabilities, making it challenging to achieve optimal detection without encountering a significant number of false positive results. Due to the varied size and blurred boundaries of BMs, developing an automatic algorithm to reduce false positive results while maintaining excellent sensitivity is quite challenging. In view of this challenge, a modified YOLOv5 called SA-YOLOv5 (self-attention YOLOv5) is proposed in this study.

On the basis of YOLOv5, SA-YOLOv5 adds an attention mechanism and transformer block to enhance the features of the BM region and weaken the features of vascular shadows and gray matter. For the accurate detection of small BMs, an additional prediction head is added to SA-YOLOv5. Meanwhile, the F2-score is introduced to solve the difficulty of determining the confidence threshold to ensure more appropriate detection performance. To demonstrate the detection ability of SA-YOLOv5, we compared it with four existing methods on the internal testing set and validated the method’s effectiveness and generalizability on the external testing set.

## 2. Materials and Methods

### 2.1. Study Participants

**Figure 1:**
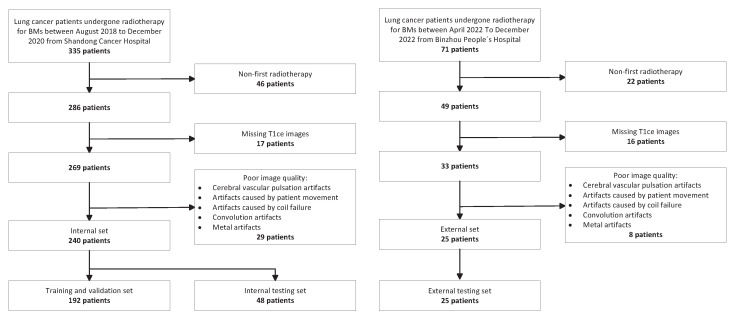
Flowchart of patient inclusion and exclusion criteria.

Approval to carry out this study was received from the institutional review board, and the identification information of the enrolled patients was anonymized; the requirement to obtain written informed consent was waived due to the retrospective nature of the study. In all, 335 lung cancer patients who underwent radiotherapy for BMs at Shandong Cancer Hospital and Institute from August 2018 to December 2020 were enrolled, along with 71 lung cancer patients who underwent radiotherapy for BMs at Binzhou People’s Hospital from April 2022 to December 2022. As shown in Figure 1, after patient exclusion, a total of 240 and 25 patients were finally included in the internal set and external set, respectively. All patients in the internal set were imaged using a GE Discovery MR 750W scanner with six channels of head coils in the same posture. The patients in the external set were all imaged using a Siemens Magentom Avanto scanner.

### 2.2. Dataset Construction

**Figure 2:**
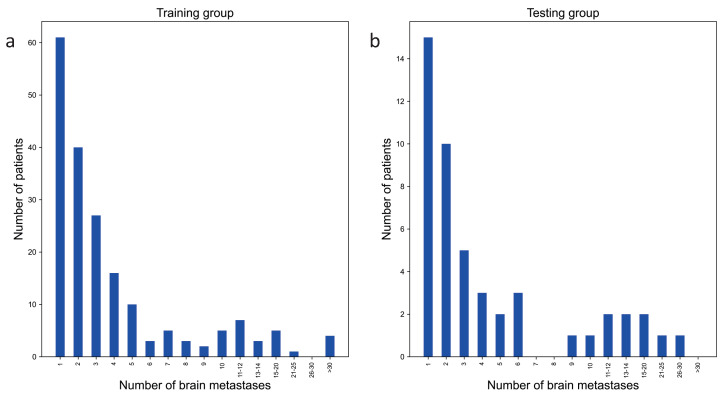
Distributions of BMs in the training group and internal testing set: (**a**) distribution of BMs in the training group and (**b**) distribution of BMs in the internal testing set.

All these patients had complete single-sequence T1-weighted contrast-enhanced (T1ce). The MRI images were manually annotated by two oncologists with five and six years of experience, respectively, utilizing MIM Maestro 6.8.2 software. Each oncologist annotated all images, while another oncologist with sixteen years of experience modified the mask if necessary and confirmed any inconsistent areas. There were a total of 1030 BMs in the internal dataset, for average of 4.3 BMs per patient. The external set included 130 BMs, for an average of 5.2 BMs per patient. The patients in internal set were randomly divided into a training group and a testing group at a ratio of 8:2 (192:48). To make the SA-YOLOv5 framework focus on the characteristics of BM regions, the slices of patients without BM regions were eliminated in the training group, preserving 2269 images. These preserved images were divided into a training set and a validation set at a ratio of 8:2. The validation set was used to verify the model’s performance and save the best weights of the DL method. The internal testing set used for model evaluation consisted of whole MRI images from the 48 patients in the testing group. The respective distributions of BMs in the training group and internal testing set are shown in Figure 2.

**Figure 3:**
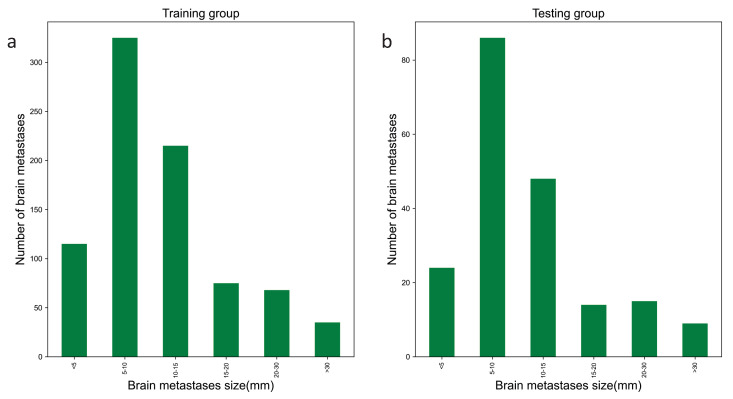
The distribution of BM maximum diameters in the training group and internal testing set: (**a**) distribution of BM maximum diameters in the training group and (**b**) distribution of BM maximum diameters in the internal testing set.

The mask of each BM was projected based on the transverse section direction and the maximum diameter of each BM was determined by calculating the minimum circumcircle of the projection using the opencv_python (version 4.2.0) software package while considering the pixel scale with assistance from the pydicom (version 2.2.0) software package. As shown in Figure 3, the distribution of BM maximum diameters is relatively concentrated. The proportion of BMs with a maximum diameter of 0–10 mm is 55.8%. Small BMs typically have poorly defined boundaries and low contrast. In regular item training, the precision (*P*) with respect to small targets is generally much lower than that for medium and large targets. Moreover, the distribution of small BMs is not uniform. These two issues complicate BM detection.

### 2.3. Dataset Ground Truth

The bounding box for each BM had a unique coordinate format produced from the corresponding segmentation masks. In order to make the bounding box properly represent the ROI of BM, an iterative algorithm was used to find the outermost pixel values of the segmentation mask to build the bounding box, making the produced bounding boxes were reliable enough to complete the training and testing of the proposed model.

### 2.4. Image Preprocessing

The MRI images were cut into 384×384 slices and processed via mosaic data enhancement. The mosaic data augmentation technique utilizes random scaling, cropping, and arrangement to splice four images into a single large image [18]. The advantages of this method are that the background of the image is enriched and the batch size is increased through concatenation of the four original images.

### 2.5. Architecture of SA-YOLOv5

**Figure 4:**
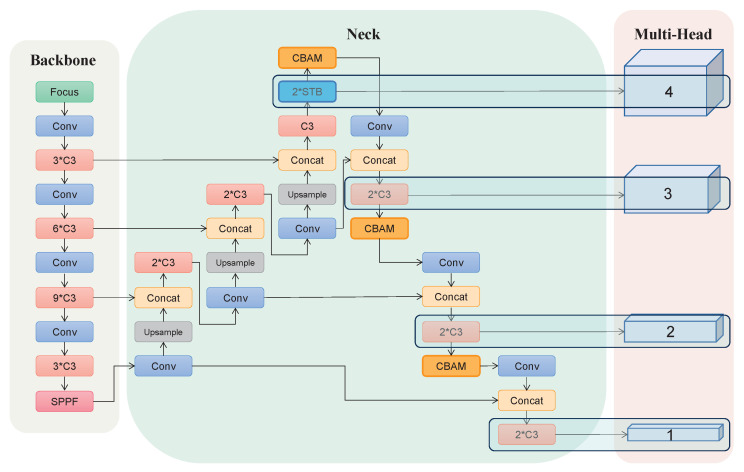
The architecture of SA-YOLOv5. CBAM: convolutional block attention module, STB: Swin transformer block, SPPF: spatial pyramid pooling–fast. Conv is composed of a 1×1 convolutional block, batch normalization unit [22], and SiLU (sigmoid-weighted linear unit) [23].

As illustrated in Figure 4, the main architecture of SA-YOLOv5, including the backbone, neck, and multi-head, was implemented by using Pytorch (https://pytorch.io (accessed on 1 October 2022)) [19]. The proposed SA-YOLOv5 utilized in this paper made the following improvements to YOLOv5 [14]. First, an additional prediction head was added for more accurate detection of small BMs. Second, a self-attention block [20] was introduced to enhance the channel of the effective features. Third, a Swin transformer block [21] was added to strengthen the attention to small targets on the feature map. The main modules in the SA-YOLOv5 architecture are described in detail below.

#### 2.5.1. Backbone

**Figure 5:**
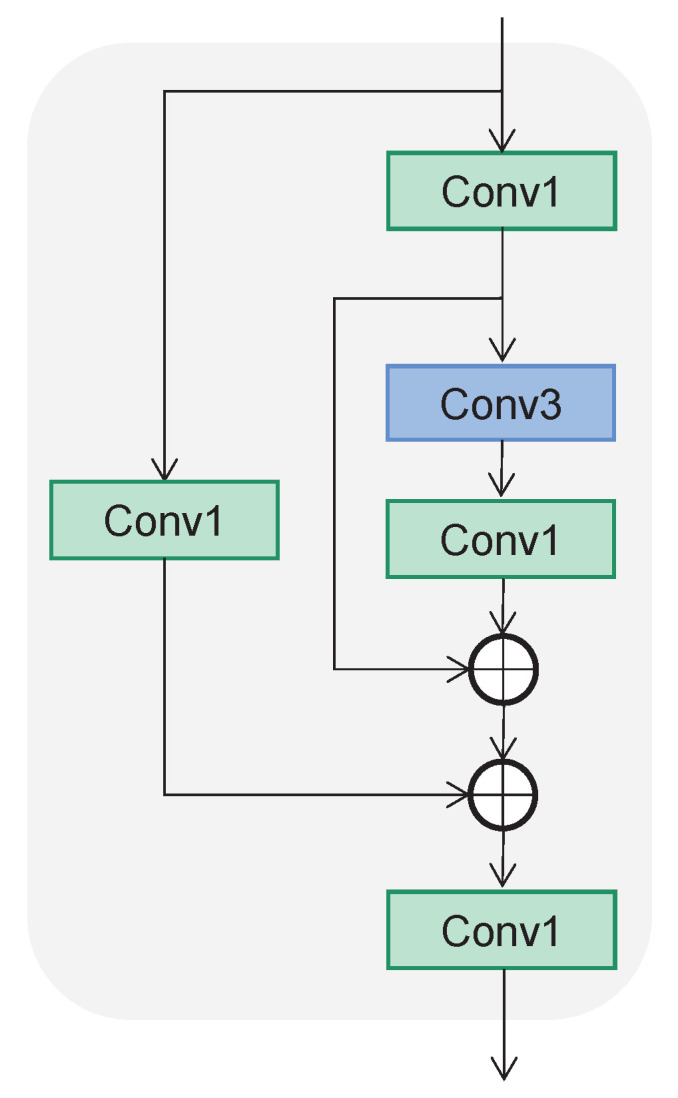
The structure of C3 block. The Conv1 block is composed of a 1×1 convolutional block, batch normalization unit, and SiLU. The Conv3 block is composed of a 3×3 convolutional block, batch normalization unit, and SiLU.

**Figure 6:**
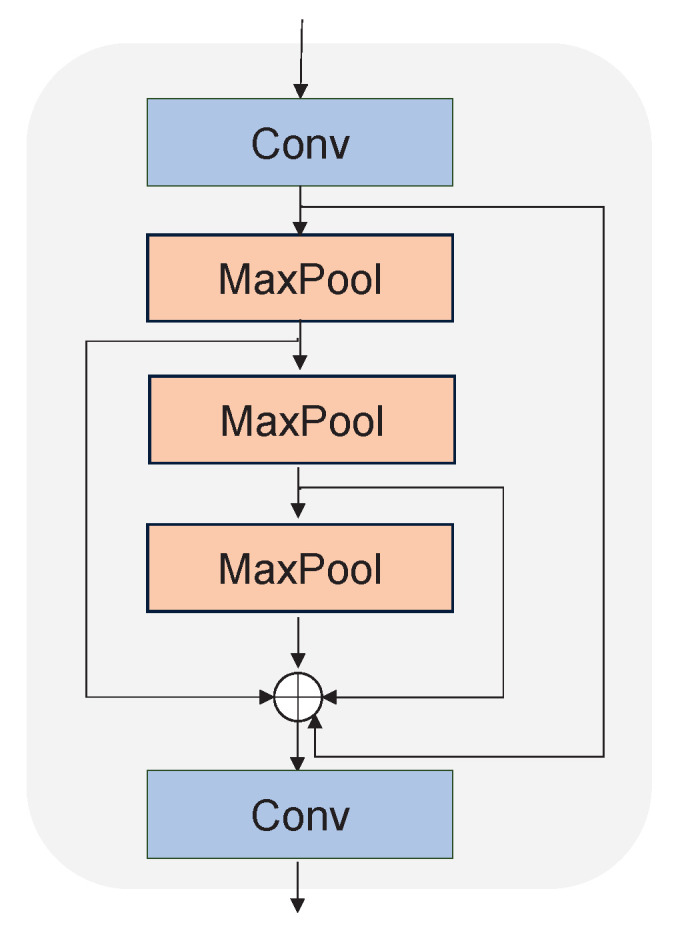
The structure of the SPPF block. The Conv block is composed of a 1×1 convolutional block, batch normalization unit, and SiLU.

The backbone consists of a Focus block, Conv block, C3 block, and SPPF block. The Focus block is implemented using a 6×6 convolutional block, while the Conv block is composed of a 1×1 convolutional block, batch normalization unit, and SiLU. As shown in Figure 5, the C3 block is constructed via two residual structures composed of several convolutional blocks. The Conv1 block is composed of a 1×1 convolutional block, batch normalization unit, and SiLU, while the Conv3 block is composed of a 3×3 convolutional block, batch normalization unit, and SiLU. Furthermore, the SPPF block, shown in Figure 6, consists of the Conv block and MaxPool layers. The feature maps output by the MaxPool layers are concatenated together and then reshaped by the Conv block.

#### 2.5.2. Neck

As shown in Figure 4, a path aggregation network (PAN) [24] structure is added to the neck in combination with the feature pyramid network (FPN) [25] structure to form a powerful feature fusion layer in the neck. Furthermore, a convolutional block attention module (CBAM) [20] is added to the neck to capture important information on small BMs. The CBAM contains a Channel Attention Module (CAM) and a Spatial Attention Module (SAM), which can be mathematically expressed as

(1) $$M_{c}\left(F\right)=\sigma \left(MLP\left(AvgPool\left(F\right)\right)+MLP\left(MaxPool\left(F\right)\right)\right)$$

(2) $$M_{s}\left(F\right)=\sigma \left(f^{7\times 7}\left(\left[AvgPool\left(F\right);MaxPool\left(F\right)\right]\right)\right)$$

where $M_{c}\left(F\right)$ and $M_{s}\left(F\right)$ respectively represent the feature maps of the channel attention map and spatial attention map, σ is the sigmoid function, AvgPool and MaxPool respectively represent the global average pooling and global max pooling, F denotes the input feature map, MLP denotes multi layer perceptron, and $f^{7\times 7}$ denotes the convolutional block with a filter size of 7×7.

**Figure 7:**
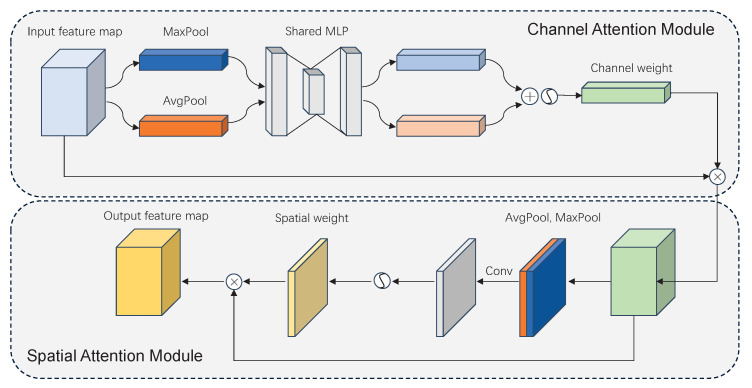
The structure of CBAM, consisting of the CAM and SAM.

The CBAM derives the attention map from the given feature map along the channel and space dimensions, then multiplies the attention map by the input feature map for adaptive feature refinement, thereby enhancing the contribution of informative feature channels and weakening the interference of useless channels. The CAM plays a crucial role in integrating relevant information during the merging process of the feature map. As shown in Figure 7, the feature map initially undergoes maximum pooling and average pooling based on its width and height. Subsequently, a multi-layer perceptron (MLP) with shared weights is employed. The outputs from the MLP layer are summed together pixel-wise, resulting in the channel attention map $M_{c}\left(F\right)$ through activation via the sigmoid function. After the CAM, the SAM is used to focus on where the most meaningful features come from. The role of the SAM is to accurately capture and represent the spatial information inherent in the feature map. The SAM takes the output of the CAM as its input and processes it through maximum pooling and average pooling. The feature map is merged into a feature map with two channels, then passes through a 7×7 convolutional layer to reduce this to one channel, resulting the spatial attention map $M_{s}\left(F\right)$ being obtained through the sigmoid function.

#### 2.5.3. Multi-Head Attention

The prediction head in the multi-head structure is a decoder that retains significant the spatial structure information of the corresponding original image on the feature map. Its main structure is a classification layer composed of 3×3 and 1×1 convolutional blocks. As shown in Figure 4, the prediction head 1 uses the 12×12 feature map as input; an element in the 12×12 feature map corresponds to a pixel region of 32×32 in the 384×384 input image. Accordingly, the element in the feature map of the prediction head 2 and 3 corresponds to a pixel region of 16×16 and 8×8, respectively. In order to effectively detect small BMs, we added an additional small object prediction head combining a Swin transformer block (STB) [21] and 1×1 convolutional blocks, which is called a Swin transformer prediction head (STPH). The prediction head 4 employs the 96×96 feature map as input, and every element in it corresponds to a 4×4 pixel region of the original image.

**Figure 8:**
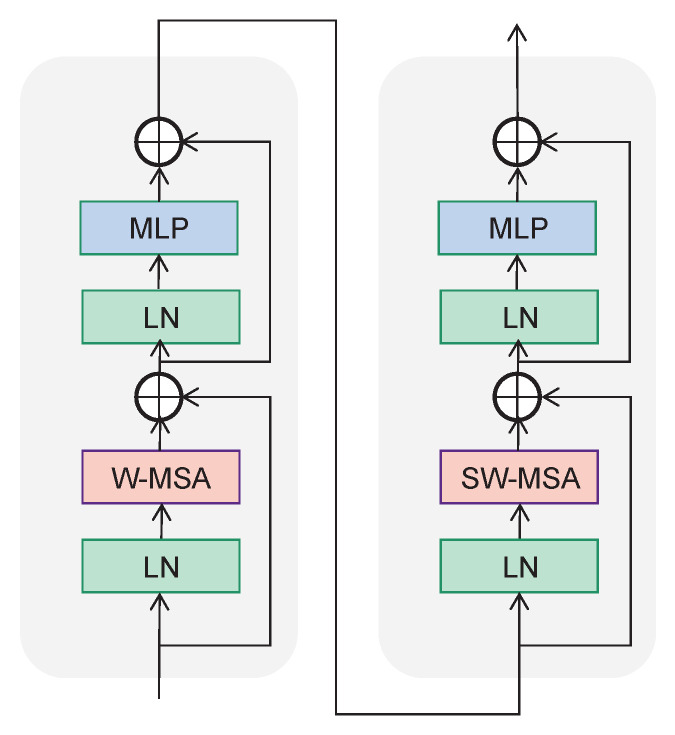
The architecture of the Swin transformer block. LN: layer normalization, MLP: multilayer perceptron, W-MSA: windows multi-head self-attention, SW-MSA: shifted windows multi-head self-attention.

**Figure 9:**
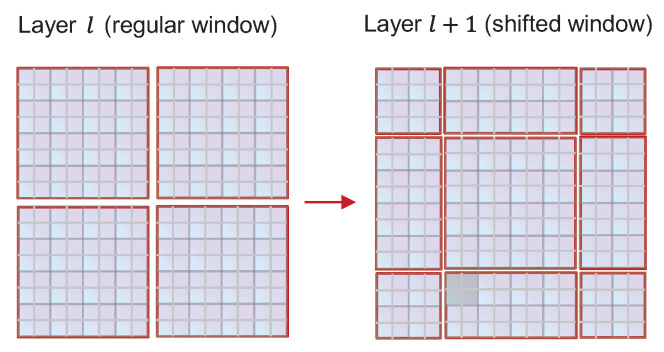
Windows used for self-attention computations in the adjacent layers l and l plus one are divided into a regular window and shifted window.

Unlike the common multi-head self attention module, the STB is formed based on the concept of shifted windows [26]. As shown in Figure 8, two consecutive blocks are in a string connection, with each block is composed of a multilayer perceptron (MLP) with a SiLU, LayerNorm (LN) layer, and multi-head self attention module. The windows multi-head self-attention (W-MSA) module and shifted windows multi-head self-attention (SW-MSA) module are respectively contained in these two blocks. The STB employs W-MSA and SW-MSA to change the regular window partitioning method, instead using the more efficient shifted window partitioning strategy shown in Figure 9. Not only does this maintain efficient computation of non-overlapping windows, it establishes connections between different windows. In the W-MSA module, the feature map is divided into 8×8 windows and the self-attention operation is performed within each window. The purpose of the SW-MSA module is to realize information exchange between different windows. The attention window shifts between different attention heads, meaning that each attention head focuses on a different window, thereby increasing the diversity of features. The STB can be mathematically expressed as follows:

(3) $${\hat{a}}^{l}=W-MSA\left(LN\left(a^{L-1}\right)\right)+a^{L-1}$$

(4) $$a^{l}=MLP\left(LN\left({\hat{a}}^{l}\right)\right)+{\hat{a}}^{l}$$

(5) $${\hat{a}}^{l+1}=SW-MSA\left(LN\left(a^{l}\right)\right)+a^{l}$$

(6) $$a^{l+1}=MLP\left(LN\left({\hat{a}}^{l+1}\right)\right)+{\hat{a}}^{l+1}$$

where ${\hat{a}}^{l}$, $a^{l}$ represent the outputs of the self-attention module and the MLP module of the $l^{th}$ block, respectively.

Unlike the normal multi-head self-attention computation, the relative position bias term *B* is included in the self-attention calculation of each head. The self-attention is computed as follows:

(7) $$Attention\left(Q,K,V\right)=Softmax\left(\frac{QK^{T}}{\sqrt{d}}+B\right)V$$

where *Q*, *K*, *V*, and *B* respectively denote the query, key, value, and bias matrices in the self-attention mechanism.

### 2.6. Boundary Loss Function

**Figure 10:**
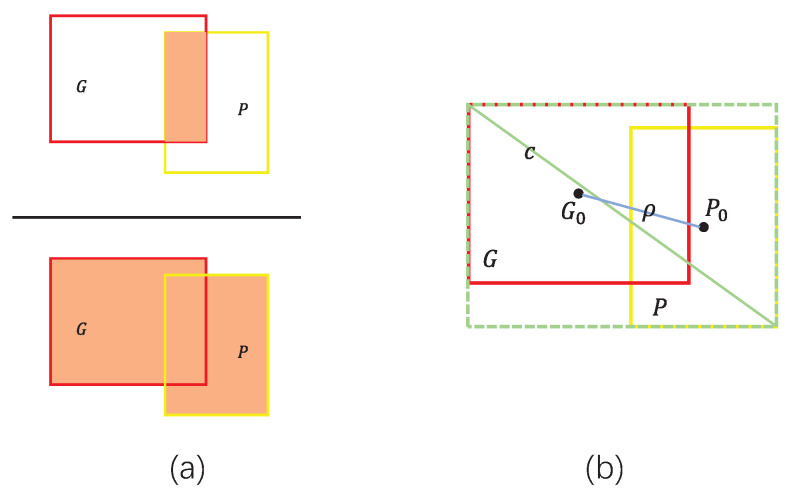
(**a**) IoU schematic and (**b**) schematic diagram of CIoU penalty items. Here, $P_{0}$ and $G_{0}$ represent the respective center points of *P* and *G*, ρ is the Euclidean distance between $P_{0}$ and $G_{0}$, and *c* denotes the diagonal length of the minimum outer rectangle between *P* and *G*.

The most common method for punishing imprecise prediction bounding boxes is to employ the Intersection over Union (IoU) loss function [27] or its improved variant. As shown in Figure 10a, the IoU is used to quantify the ratio of the intersection and concatenation of prediction bounding box (*P*) and ground truth bounding box (*G*). The IoU and $Loss_{IoU}$ can be expressed as follows:

(8) $$IoU=\frac{\left|P\cap G\right|}{\left|P\cup G\right|}$$

(9) $$Loss_{IoU}=1-IoU$$

where *P* and *G* denote the prediction bounding box and the ground truth bounding box, respectively, and $Loss_{IoU}$ is the IoU loss function.

When *P* and *G* do not intersect and IoU(P,G)=0, the gradient cannot be transferred back and the $Loss_{IoU}$ cannot optimize this situation. To address this flaw, the Complete IoU (CIoU) loss function [28] is utilized for bounding box regression, leading to faster convergence and better performance than $Loss_{IoU}$. The CIoU, as illustrated in Figure 10b, introduces a penalty term $R=\frac{\rho^{2}}{c^{2}}+\alpha v$ such that the IoU, center point Euclidean distance, and aspect ratio are included in the consideration range. The $Loss_{CIoU}$ can be mathematically expressed as follows:

(10) $$Loss_{CIoU}=1-IoU+\frac{\rho^{2}}{c^{2}}+\alpha v$$

(11) $$v=\frac{4}{\pi^{2}}{\left(arctan\frac{w_{G}}{h_{G}}-arctan\frac{w_{P}}{h_{P}}\right)}^{2}$$

(12) $$\alpha =\frac{v}{1-IoU+v}$$

where α denotes the weight parameter of *v*, *v* is used to measure the similarity of the aspect ratio of *P* and *G*, ρ is the Euclidean distance between $P_{0}$ and $G_{0}$, *c* represents the diagonal length of the minimum outer rectangle between *P* and *G*, and $w_{G}$, $w_{P}$, $h_{G}$, and $h_{P}$ respectively denote the widths and heights of *G* and *P*.

### 2.7. Training Configuration and Procedure

Data augmentation was only performed on the training set. The validation set and testing set usd the original data. The weights of the convolutional kernels were initialized by loading the pretraining weight file of the YOLOv5 model. The CIoU loss function [28] was selected as the penalty measurement index of the bounding boxes. An SGD (stochastic gradient descent) optimizer [29] was used to train the network. The initial learning rate was set to 0.01 and reduced by 10% if the validation loss did not improve after three epochs. The training procedure was terminated if the validation loss did not improve within twenty epochs. The model was trained on a Geforce GTX 1080Ti with a memory of 11 GB (NVIDIA, Santa Clara, CA, USA).

### 2.8. Postprocessing

The output prediction results from the prediction heads are processed by means of non-maximum suppression (NMS) [30]. The basic idea behind NMS is to select the bounding box with the highest confidence score and remove all other bounding boxes that have a significant overlap with it. This is repeated for all remaining bounding boxes until there are no more overlaps. The prediction results after NMS represent the final output of the model.

### 2.9. Model Evaluation

Each bounding box generated by the detection algorithm is accompanied by a confidence score that ranges from 0% to 100%. The confidence threshold is used to ignore bounding boxes with a confidence value less than the confidence threshold. Previous BM detection studies have used a confidence threshold of 50% [9,31,32] or confidence thresholds ranging from 0.1 to 0.9 [11]; however, these approach may not lead to optimal results for BM detection. Other object detection research has utilized the F1-score, which represents the harmonic mean of precision and recall, to determine the optimal confidence threshold [33,34,35]. As the recall is more important than the precision in BM detection, we introduced the F2-score, which emphasizes the importance of recall by assigning it twice the weight of precision, to determine the optimal confidence threshold.

Although DL detection algorithms for slice-by-slice prediction have been developed, the final performances of these models is evaluated in three-dimensional space. The neighboring bounding boxes of the final prediction are stacked to form a volume, which is considered to be a true positive result if it overlaps with the ground truth. On the other hand, the prediction volume is considered a false positive result if there is no voxel overlap with the ground truth volume.

The evaluation metrics adopted in this study included recall (*R*), precision (*P*), and F2−score. The precision is the proportion of the number of true positive results to the number of all predicted results in the testing set. The recall is the proportion of the number of true positive results to the number of all BMs in the testing set. These evaluation metrics were defined as follows:

(13) $$P=\frac{TP}{TP+FP}$$

(14) $$R=\frac{TP}{TP+FN}$$

(15) $$F2-score=\frac{5\times P\times R}{4\times P+R}$$

where TP, FP, and FN represent the number of true positives, false positives, and false negatives, respectively.

## 3. Results

In this section, the prediction results of our proposed model and other state-of-the-art deep learning models (Faster R-CNN [36], SSD [9], FF-SSD [11], and EfficientDet [37]) are compared on the testing set. During our experiments, we strictly followed the principle of randomization when partitioning the dataset. In addition, the dataset was partitioned based on patients rather than on individual BMs, resulting in a more varied distribution of the number and size of BMs in the training set and testing set. This approach produces a testing environment that is closer to the clinical situation.

### 3.1. Detection Performance of SA-YOLOv5

**Figure 11:**
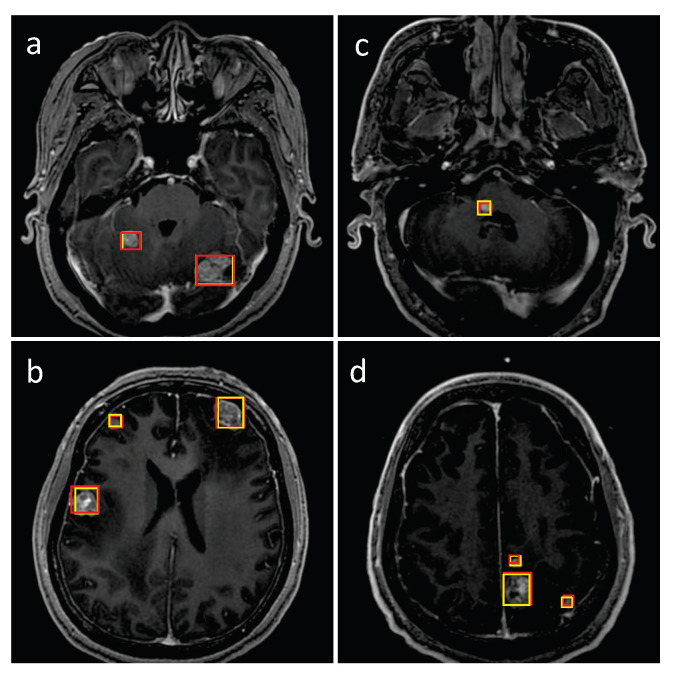
Detection performance of the proposed model on internal testing MRI images: (**a**–**d**) examples of true positive inferences. The red bounding boxes represent the ground truth, while the yellow bounding boxes represent the model prediction results.

**Figure 12:**
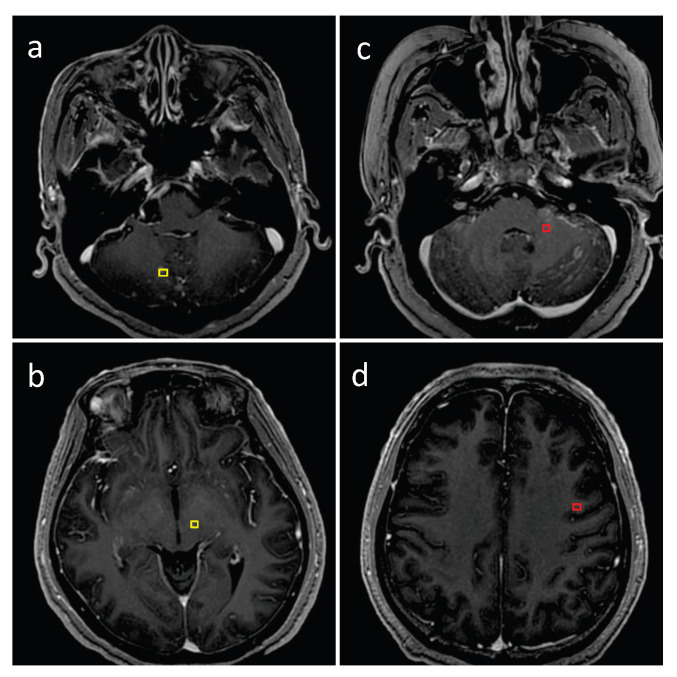
Detection performance of the proposed model on internal testing images: (**a**,**b**) examples of false negative inferences and (**c**,**d**) examples of false positive inferences. The red bounding boxes represent the ground truth, while the yellow bounding boxes represent the model prediction results.

The representative testing images in Figure 11 and Figure 12 show true positive, false positive, and false negative inferences from the proposed model. The red bounding boxes represent the ground truth, while the yellow bounding boxes represent the model prediction results. As shown in Figure 11 and Figure 12, the proposed SA-YOLOv5 model can detect almost all BMs with regular volumes. However, blood vessels and calcification in MRI images can cause interference during the BM detection process, resulting in false negative and false positive results.

### 3.2. Comparison with Existing Detection Methods

Table 1 lists the results of the proposed model and four existing models used for comparison. From the table, it can be seen that the recall achieved by Faster R-CNN [36] is 0.690, which is 0.348 higher than its precision. As Faster RCNN is a two-stage detector, its detection speed is relatively slow. Meanwhile, there is a contradiction between semantics and space, making it more difficult to achieve a good balance between deep and shallow feature maps. EfficientDet [37] is the only model with a recall lower than its precision, achieving a precision of 0.669 and recall of 0.614. In EfficientDet, the feature map’s receptive field of feature points is much larger than the downsampling rate. As a result, there are many features from surrounding regions at the points of the feature map; small BMs occupy fewer features, which affects the BM detection results. The performance evaluation metrics of SSD were imbalanced as well, with a recall of 0.822 and precision of 0.369. Although SSD uses multi-layer feature maps, the semantic information of the shallow feature maps is insufficient and feature fusion is not carried out, resulting in poor BM detection results. In contrast to SSD [9], FF-SSD [11] adds a multi-level feature fusion technique to enhance the feature maps of small object, resulting in higher precision and more true positive results. Overall, while SSD and FF-SSD exhibit high accuracy in identifying BMs, they have limitations due to false positive results. Among all the models, our proposed model achieves a recall of 0.904 and precision of 0.612. This suoerior performance may be attributed to the effectiveness of the proposed preprocessing techniques and feature fusion layer. Obviously, the detection results focused more on the recall and slightly sacrifice the precision, as this is more suitable for clinical applications in auxiliary diagnosis.

**Figure 13:**
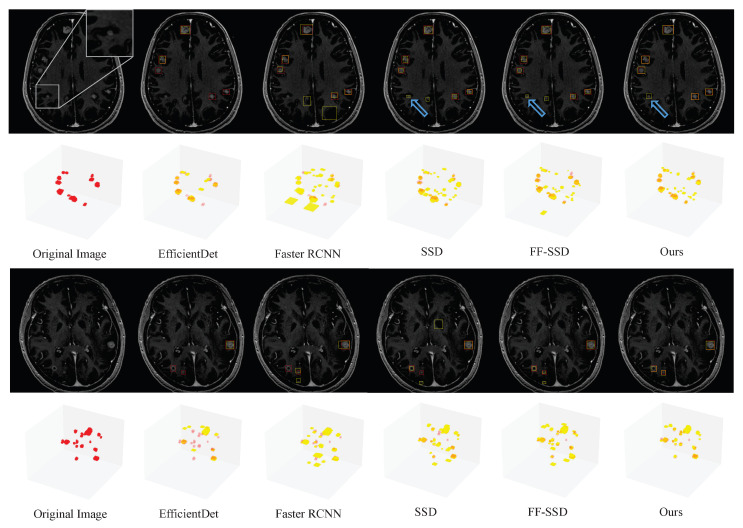
Ground truth and prediction results of the different models on original MRI images of two distinct patients. The upper photos show 2D visualizations and the lower photos show 3D visualizations. The red bounding boxes represent the ground truth, while the yellow bounding boxes represent the model prediction results. The arrow indicates the possible lesion with no ground truth.

To more intuitively compare the performance of the different detection models, Figure 13 displays the ground truth and prediction results on the original MRI images of two distinct patients. The predicted results of these two patients are visualized in 2D and 3D, respectively. The red bounding boxes represent the ground truth, while the yellow bounding boxes represent the model prediction results. Notably, EfficientDet [37] and Faster R-CNN [36] do not perform well in detecting BMs. EfficientDet [37] generates fewer prediction bounding boxes, resulting in many undetected lesions. Conversely, Faster R-CNN identifies many vascular regions as BMs, leading to a high number of false positive results. Compared with EfficientDet [37] and Faster RCNN [36], SSD [9], FF-SSD [11], and our proposed model all demonstrate superior detection performance. In particular, our proposed model successfully detects all the BMs distributed in the original images, with arrows indicating possible lesions around the yellow bounding box. A magnified view of this region is visible in the upper right corner of the original image. When re-examined by two oncologists and one physicist, this was revealed to be an undiscovered BM with similar characteristics to those of blood vessels. Compared to other models, SA-YOLOv5 does not misidentify so many vascular regions as BMs, and shows accurate identification ability.

### 3.3. Effectiveness Analysis of the Improvements Made in SA-YOLOv5

To verify the effectiveness of the improvements made in SA-YOLOv5, the prediction performance of YOLOv5 [14], YOLOv5 + CBAM, YOLOv5 + CBAM + PH, and YOLOv5 + CBAM + STPH are compared in this section. The precision, recall, F2-score, FN per patient, and FP per patient of these models are listed in Table 2. It can be seen from the table that the CBAM block added to the neck structure improved the recall from 0.883 to 0.898, while the precision was decreased slightly. The model produced 177 true positive results and 119 false positive results. These results indicate that CBAM pays more attention to the boundary information of BMs, effectively increasing true positive results while resulting in the misidentification of a small number of vascular areas as BMs. The inclusion of the additional prediction head (STPH without STB) led to an increase in the model’s bounding box output; however, it did not yield improved prediction results. Moreover, it treated a number of small calcification or vascular structures as BMs. The addition of STB enhances the detection ability. The STPH layer is regarded as a detection layer for extremely small BMs, and effectively strengthens the distinction between BMs and the background. YOLOv5 + CBAM + STPH achieved state-of-the-art performance, increasing the recall by 2.1% while ensuring that the precision remained almost unchanged from the baseline. Moreover, it produced the most true positive results. These results indicate that the addition of the CBAM and STPH can effectively improve the performance of YOLOv5 on BM detection.

### 3.4. Detection Performance on the External Testing Set

**Figure 14:**
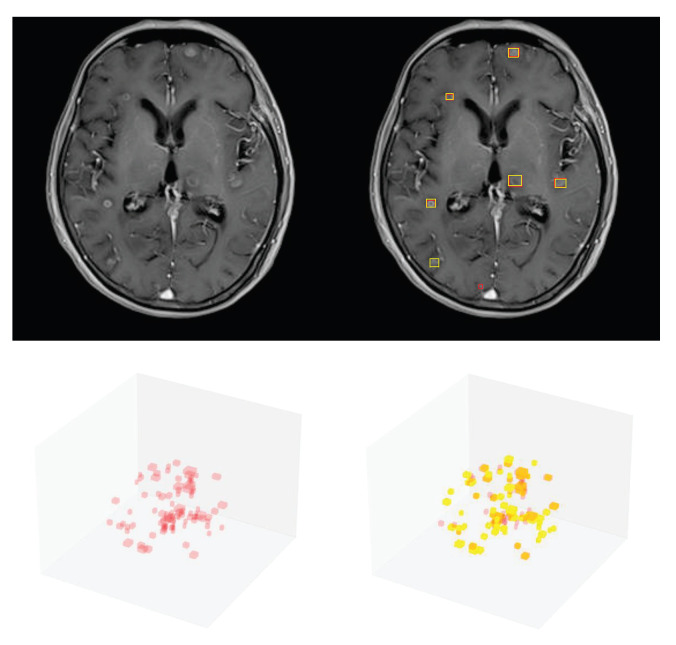
Prediction results of our proposed model on the external set. The photos show 2D and 3D visualizations of the same patient. The red bounding boxes represent the ground truth, while the yellow bounding boxes represent the model prediction results.

To assess the proposed model’s generalizability, we evaluated it on the external testing set. Our proposed model achieved a recall of 0.854 (111/130) and precision of 0.681 (111/163) and had excellent detection results on images obtained with different scanning equipment at different institutions, demonstrating the feasibility of the proposed YOLOv5-based BM detection model. As shown in Figure 14, there were a number of false negative and false positive detection results on the external testing data. In particular, the detection of small BMs partially close to blood vessels is not effective, as these were regarded as negative samples. This may be attributed to the similarity in characteristics between these types of BMs in the external testing data and calcification structures in the training data. In addition, the better precision observed on the external testing data may be attributed to the relatively higher contrast of these images compared to the internal data.

## 4. Discussion

The improved YOLOv5 algorithm proposed in this paper, called SA-YOLOv5, shows promising results in assisting detection of NSCLC brain metastases. The performance of the proposed model was rigorously evaluated on the internal and external testing sets. Our experimental results show that SA-YOLOv5 can detect nearly all BMs with maximum diameters of 0.5 cm or larger while limiting false positive results. The proposed SA-YOLOv5 achieved a precision of 0.612 and a recall of 0.904 on the internal testing set. Compared to the compared methods, our model demonstrates a significant improvement in both recall and precision. Furthermore, upon evaluating the detection results with two oncologists and one physicist, we discovered three small BMs that were initially overlooked. Our proposed model maintained its BM detection ability on the external testing set, achieving a precision of 0.681 and a recall of 0.854. Overall, SA-YOLOv5 effectively achieves balanced performance in auxiliary diagnosis while avoiding interference from false positive results.

This study presents several limitations that are manifested in the dataset and detection performance. The quality of MRI images varies due to differences in scanning equipment and the parameters used during scanning. Additionally, the sample distribution of patients in different regions may have an impact on the testing results. In future work, continuing to collect MRI data for model training from multiple institutions and scanners represents an effective method to improve the model performance varied data. While the proposed model shows enhanced detection capacity for small BMs, but it can nonetheless generate false negative and false positive results for such lesions. Additionally, the quality of dataset delineation imposes certain restrictions on the model. Even though the delineation outcomes were reviewed by multiple radiologists, BMs may have been missed. The model could be further refined by incorporating additional MRI data on BMs in order to facilitate the detection of exceedingly small BMs and further improve the model’s generalization and robustness.

The current DL models for the segmentation of multiple BMs exhibit considerable scope for improvement in both performance and generalization ability. In addition to automatic detection, future research might focus on developing end-to-end segmentation approaches based on DL. To this end, doctors’ diagnostic experience should be incorporated as prior knowledge to constrain the model and optimize its loss function. Ultimately, an automated BM detection and segmentation model with high accuracy and speed can greatly enhance radiologists’ work efficiency.

## 5. Conclusions

In this paper, we propose a novel BM detection algorithm based on a self-attention mechanism, which we call SA-YOLOv5. To explore the potential information of the feature map, we employ the CBAM and STPH to capture the features of small BMs. Our experimental results demonstrate that the proposed model attains comparatively well-rounded BM detection performance and that the CBAM and STPH effectively improve the performance of YOLOv5. We believe that our proposed framework can be used as a reliable computer-aided diagnosis system for BM detection using T1ce.

## Figures and Tables

**Table 1:** Prediction results of different deep learning models on the internal testing set.

Method	Recall	Precision	F2-Score	FN/Patient	FP/Patient
Faster R-CNN [36]	0.690(136/197)	0.342(136/398)	0.573	1.271	5.458
EfficientDet [37]	0.614(121/197)	0.669(121/181)	0.624	1.583	1.250
SSD [9]	0.822(162/197)	0.369(162/439)	0.660	0.729	5.771
FF-SSD [11]	0.827(163/197)	0.397(163/411)	0.680	0.708	5.167
Ours	0.904(178/197)	0.612(178/291)	0.825	0.396	2.354

**Table 2:** The prediction results of our proposed model and its variants on the internal testing set.

Model	Recall	Precision	F2-Score	FN/Patient	FP/Patient
YOLOv5	0.883(174/197)	0.611(174/285)	0.812	0.479	2.313
YOLOv5+CBAM	0.898(177/197)	0.598(177/296)	0.816	0.417	2.479
YOLOv5+CBAM+PH ^1^	0.898(177/197)	0.586(177/302)	0.812	0.417	2.604
YOLOv5+CBAM+STPH	0.904(178/197)	0.612(178/291)	0.825	0.396	2.354

^1^ PH denotes the prediction head 4 without STB.

## Data Availability

In order to safeguard the confidentiality of the participants, the data pertaining to this study are currently withheld from public access. The data can be shared upon request.

## Abbreviations

The following abbreviations are used in this manuscript:

BM	Brain metastasis
DL	Deep learning
NSCLC	Non-small-cell lung cancer
SRS	Stereotactic radiosurgery
ROI	Region of interest
SSD	Single-shot detector
PPV	Positive predictive value
FF	Feature fusion
YOLOv5	You only look once version 5
SA-YOLOv5	Self-attention YOLOv5
T1ce	T1-weighted contrast-enhanced
P	Precision
R	Recall
CBAM	Convolutional block attention module
CAM	Channel Attention Module
SAM	Spatial Attention Module
STB	Swin transformer block
STPH	Swin transformer prediction head
SW-MSA	Shifted windows multi-head self-attention
W-MSA	Windows multi-head self-attention
NMS	Non-maximum suppression
IoU	Intersection over union
SGD	Stochastic gradient descent
